# Diffraction anisotropy falloff in the direction of the detergent belt for two centered monoclinic crystals of OmpF

**DOI:** 10.1016/j.dib.2016.03.031

**Published:** 2016-03-11

**Authors:** Vincent Chaptal, Arnaud Kilburg, David Flot, Benjamin Wiseman, Nushin Aghajari, Jean-Michel Jault, Pierre Falson

**Affiliations:** a Drug Resistance Mechanism and Modulation team, Institut de Biologie et Chimie des Protéines (IBCP), UMR5086 CNRS/Université Lyon 1, 7 passage du Vercors, F-69367 Lyon, France; b ESRF – The European Synchrotron, 71 Avenue des Martyrs, Grenoble, France; c Université Grenoble Alpes, Institut de Biologie Structurale (IBS), CNRS UMR 5075, 6 rue Jules Horowitz, F-38027 cedex-1 Grenoble, France; d CEA, DSV, IBS, F-38000 Grenoble, France; e Biocrystallography and Structural Biology of Therapeutic Targets team, Institut de Biologie et Chimie des Protéines (IBCP), UMR5086 CNRS/Université Lyon 1, 7 passage du Vercors, F-69367 Lyon, France

**Keywords:** Diffraction anisotropy, Membrane protein

## Abstract

This data article describes the anisotropy of diffraction observed for the centered monoclinic crystals of OmpF reported in “Two different centered monoclinic crystals of the *E. coli* outer-membrane protein OmpF originate from the same building block (Chaptal et al., 2016 [1])”. The datasets intensity falloff as a function of resolution are provided along with reflections along the (h,l) and (k,l) planes. A comparison with the crystal packing in the real cell is also provided, with the correspondence to the reciprocal vectors. These data can be retrieved from the Protein Data Bank under accession codes PDB: 4jfb and PDB: 4d5u.

**Specifications Table**

**Table:** 

Subject area	*Biology*
More specific subject area	*Crystallography*
Type of data	*Figure*
How data was acquired	*X-ray diffraction of crystals*.
Data format	*Analyzed*
Experimental factors	*Comparison of the direction of diffraction anisotropy in reciprocal space and lack of protein:protein contact in real space.*
Experimental features	*The reflection files were subjected to anisotropy analysis using the UCLA anisotropy server*, *and simulated diffraction images along the h=0 and k=0 were generated in the Phenix package.*
Data source location	*Data for the crystal in C2 with tNCS were collected at the ESRF*, *and data in I2 were collected at ALBA synchrotrons, respectively.*
Data accessibility	*Data is within this article*. *Protein Data Bank under accession codes PDB: 4jfb and PDB: 4d5u*.

**Value of the data**

• This report is the first correspondence between diffraction anisotropy in reciprocal space and crystal packing in real space for a membrane protein.
• A detailed explanation of how this comparison is made provides a mean for future investigators to easily reproduce this analysis of their diffraction data.

## 1 Data

**Fig. 1:**
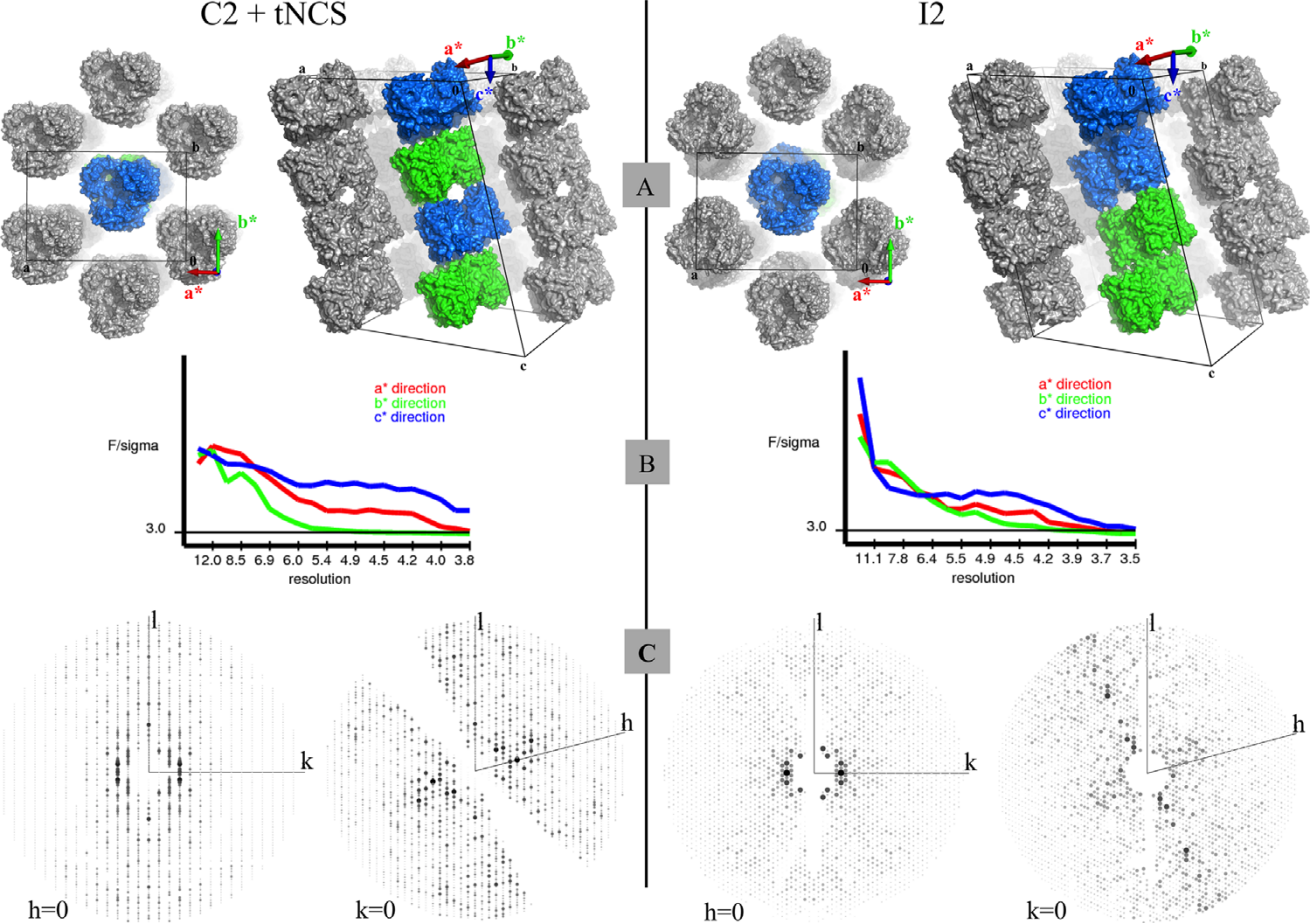
Diffraction anisotropy and crystal packing of OmpF centered monoclinic crystals. Left side: C2 with tNCS; right side: I2. **A/** The real cell is drawn in black with the origin (**O**) and the letters (**a**, **b**, **c**) symbolizing the real vectors. The reciprocal cell is outlined on the side, with vectors a*, b* and c* colored in red, green and blue, respectively, to mimic panel B. The reciprocal cell values have been determined using the online server, http://www.ruppweb.org/new_comp/reciprocal_cell.htm, and their size have been multiplied by 5000 to be able to visualize them next to the real cell. To picture crystal packing, top and side views of the two cells display OmpF in surface, with the asymmetric unit in blue, one symmetric in green and the other symmetric molecules in grey (coloring scheme similar to [1]). For the side view, the front symmetric molecules have been removed for clarity. **B/** diffraction anisotropy analysis of the whole dataset as given by the UCLA anisotropy server (http://services.mbi.ucla.edu/anisoscale/). **C/** projections of the reciprocal lattice planes *h*=0 and *k*=0 to visualize the relative diffraction strength along the three dimensions. Figures were created with Pymol [6].

In these data, diffraction anisotropy of two membrane protein crystals is presented, with an emphasis on the correspondence between real space and reciprocal space (Fig. 1). The structure corresponds to the outer porin OmpF of the Gram negative bacteria *E. coli*, for which coordinate file and structure factors can be retrieved for the d*ata in C2 with tNCS*: http://www.rcsb.org/pdb/explore/explore.do?structureId=4jfb, *and for the data in I2*: http://www.rcsb.org/pdb/explore/explore.do?structureId=4d5u.

## 2 Experimental design, materials and methods

X-ray diffraction datasets were collected at synchrotron beamlines ID23-1 [2] (ESRF) and XALOC [3] (ALBA), from flash cooled (in liquid nitrogen) single crystals and kept under nitrogen gas during data collection. Diffraction data already reduced and scaled, in the form of structure factors, were deposited in the Protein Data Bank under accession codes PDB: 4d5u and PDB: 4jfb and are freely retrievable to the community. Structure factors were submitted to the anisotropy server at UCLA for analysis (http://services.mbi.ucla.edu/anisoscale/) (Fig. 1B) [4]. The server outputs the diffraction intensity falloff as a function of resolution for the three main direction of the reciprocal space; vectors a^*^ in red, b* in green and c* in blue. The same structure factors were analyzed using the Phenix software package [5] to visualize diffraction data along the (*l,k*) and (*l*,*h*) planes to exemplify the diffraction anisotropy (Fig. 1C). *h*, *k* and *l* are the Miller indices of the reflections along the a*, b* and c* vectors, respectively. The intensity falloff is clearly seen as *k* and *h* increase, confirming a stronger diffraction along increasing *l* (c*). Fig. 1A links the real cell with the reciprocal space. The crystal packing is depicted with the asymmetric unit made of two OmpF trimers colored in blue. From the arrangement of the OmpF trimers, it is clear that most of the crystal contacts are along **c**, with the contacts along **a** and **b** being mediated by detergents, and thereby being weaker. The reciprocal space is shown with each reciprocal vector in the same color as the one used by the anisotropy server. The reciprocal cell values have been determined using the online server, http://www.ruppweb.org/new_comp/reciprocal_cell.htm, and their size have been multiplied by 5000 to be able to visualize them next to the real cell. For ease of visualization, the origin of the reciprocal cell has been offset to the right of the origin of the real cell. These data exemplify a stronger diffraction along c*, which is also the direction with the most crystal contacts, along **c**.
